# A Shifted Composition of the Lung Microbiota Conditions the Antifungal Response of Immunodeficient Mice

**DOI:** 10.3390/ijms22168474

**Published:** 2021-08-06

**Authors:** Emilia Nunzi, Giorgia Renga, Melissa Palmieri, Giuseppe Pieraccini, Marilena Pariano, Claudia Stincardini, Fiorella D’Onofrio, Ilaria Santarelli, Marina Maria Bellet, Andrea Bartoli, Claudio Costantini, Luigina Romani

**Affiliations:** 1 Department of Medicine and Surgery, University of Perugia, 06132 Perugia, Italy; emilia.nunzi@unipg.it (E.N.); rengagiorgia@gmail.com (G.R.); palmieri.melissaida@gmail.com (M.P.); marilena.pariano@gmail.com (M.P.); claudiastincardini@gmail.com (C.S.); donofrio.fiorella@libero.it (F.D.); ilasanta@libero.it (I.S.); marinamaria.bellet@unipg.it (M.M.B.); andrea.bartoli@unipg.it (A.B.); 2 University Research Center on Functional Genomics (C.U.R.Ge.F), University of Perugia, 06132 Perugia, Italy; 3 Mass Spectrometry Centre (CISM), University of Florence, 50019 Florence, Italy; giuseppe.pieraccini@unifi.it

**Keywords:** *Aspergillus*, lung microbiome, short-chain fatty acids

## Abstract

The microbiome, i.e., the communities of microbes that inhabit the surfaces exposed to the external environment, participates in the regulation of host physiology, including the immune response against pathogens. At the same time, the immune response shapes the microbiome to regulate its composition and function. How the crosstalk between the immune system and the microbiome regulates the response to fungal infection has remained relatively unexplored. We have previously shown that strict anaerobes protect from infection with the opportunistic fungus *Aspergillus fumigatus* by counteracting the expansion of pathogenic Proteobacteria. By resorting to immunodeficient mouse strains, we found that the lung microbiota could compensate for the lack of B and T lymphocytes in *Rag1^–/–^* mice by skewing the composition towards an increased abundance of protective anaerobes such as Clostridia and Bacteroidota. Conversely, NSG mice, with major defects in both the innate and adaptive immune response, showed an increased susceptibility to infection associated with a low abundance of strict anaerobes and the expansion of Proteobacteria. Further exploration in a murine model of chronic granulomatous disease, a primary form of immunodeficiency characterized by defective phagocyte NADPH oxidase, confirms the association of lung unbalance between anaerobes and Proteobacteria and the susceptibility to aspergillosis. Consistent changes in the lung levels of short-chain fatty acids between the different strains support the conclusion that the immune system and the microbiota are functionally intertwined during *Aspergillus* infection and determine the outcome of the infection.

## 1. Introduction

Commensal bacteria that inhabit the surfaces exposed to the external environment, collectively known as microbiota, engage in a symbiotic relationship with the host, and their role in health and disease is increasingly being recognized [1]. This relationship takes on particular significance in the context of defense against pathogen invasion. Indeed, the microbiota can directly interfere with pathogen colonization by competing for space and nutrients, and the local immune system can promptly respond to pathogenic threats. In parallel, the microbiota and the immune system cross-regulate each other’s activity to optimize their response against potential pathogen encounters [2,3]. For instance, the microbiota may produce immunomodulatory metabolites, such as indole-containing metabolites from the utilization of tryptophan [4], or short-chain fatty acids (SCFAs) from the fermentation of indigestible fibers [5]. SCFAs have five or less carbons and include formic acid, acetic acid, propionic acid, butyric acid, isobutyric acid, valeric acid, and isovaleric acid. SCFAs participate in a variety of physiological functions. For instance, butyrate not only represents the primary energy source for colonocytes in the gut, but also modulates the immune system, for instance by regulating the differentiation of regulatory T cells [6,7] or the activity of macrophages [8,9]. Mechanistically, butyrate is a ligand for three G protein-coupled receptors, GPR41, GPR43 and GPR109A, and works as a potent inhibitor of specific classes of histone deacetylases [10]. In turn, the microbiota can be modulated by the immune system, for instance via the release of antimicrobial peptides [11] or IgA [12]. While these mechanisms are being unraveled in the gastrointestinal tract, how the microbiota and the host immune system crosstalk in the respiratory tract to protect from infection remains less studied.

*Aspergillus* species are environmental fungi and are a potential cause of diseases in susceptible individuals, ranging from asthmatic forms to the more severe invasive aspergillosis [13]. The immune response to *Aspergillus* has been the object of intense research, and the mechanisms employed by the innate and the adaptive arms of the immune system are increasingly being elucidated [14]. It has been noted that the microbiome may also be involved in protection against *Aspergillus* [15,16]. In this regard, we have recently shown that the composition of the oropharyngeal microbiome could predict the risk of fungal pneumonia in hematological patients [17]. In particular, an increased risk of fungal pneumonia was associated with the loss of protective anaerobes such as Clostridiales and Bacteroidota and the expansion of pathogenic Proteobacteria, as confirmed in a murine model of aspergillosis [17]. However, the mechanisms at the basis of the protection as well as how the microbiota integrates with the immune system during the response to infection have not been investigated.

Based on these premises, in this study we have resorted to a murine model of *Aspergillus fumigatus* infection in immune-deficient mice, and found alterations in the composition of the lung microbiota and in the pulmonary abundance of SCFAs along characteristic patterns, supporting a role for the microbiota–immune system crosstalk in the regulation of antifungal immunity in the lung.

## 2. Results

### 2.1. Aspergillus Infection Is Associated with the Production of SCFAs

**Figure 1:**
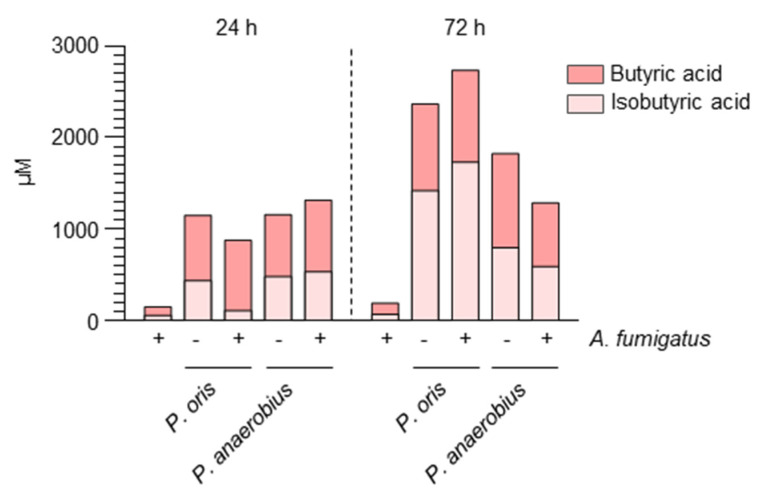
Strictly anaerobic bacteria release SCFAs in culture with *A. fumigatus*. *A. fumigatus* conidia were co−cultured in the absence or presence of either *Prevotella oris* or *Peptostreptococcus anaerobius* for 24 and 72 h, and the levels of butyric acid and isobutyric acid were determined in the medium by mass spectrometry. A representative experiment is shown.

We have previously shown that strictly anaerobic bacteria, such as *Prevotella oris* and *Peptostreptococcus anaerobius*, representative members of Bacteroidota and Firmicutes (order Clostridiales), respectively, protected against infection in a murine model of aspergillosis [17]. Since anaerobic bacteria produce SCFAs, we evaluated the presence of butyric acid and isobutyric acid in in vitro cultures and found that *Prevotella oris* and *Peptostreptococcus anaerobius* produced the two SCFAs both in the presence and in the absence of *Aspergillus* (Figure 1).

**Figure 2:**
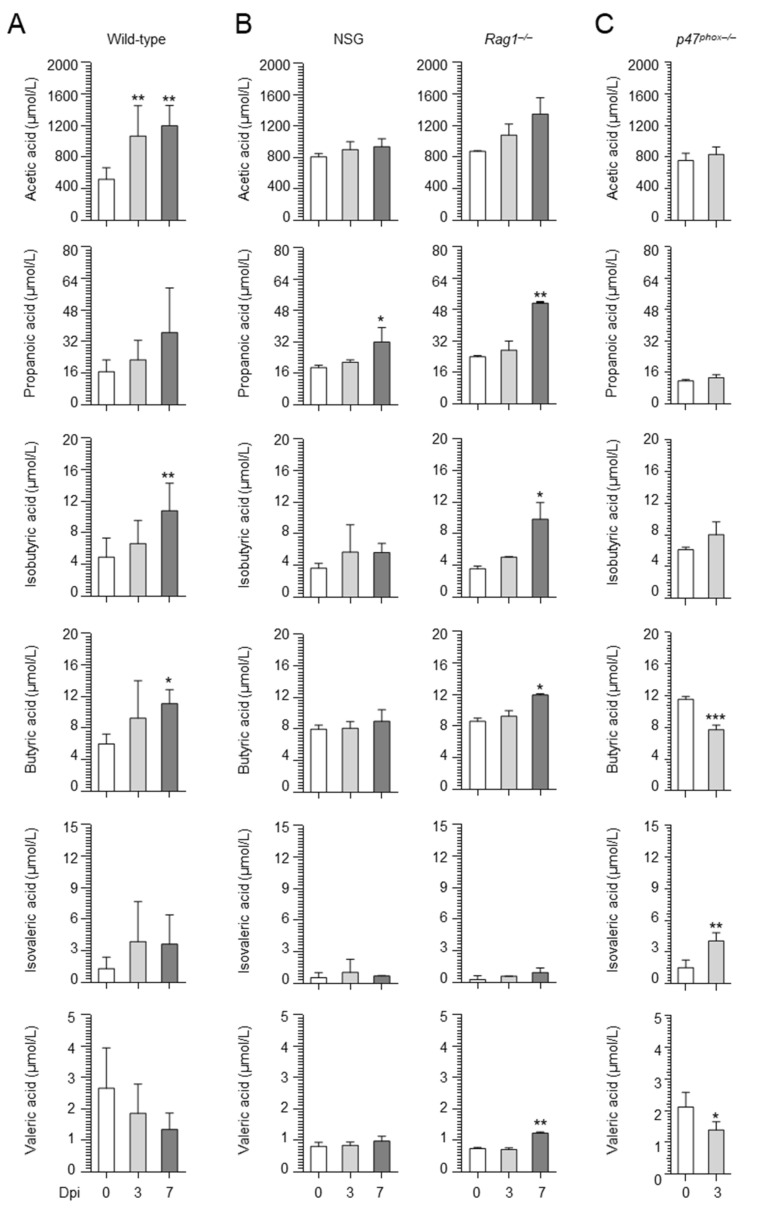
SCFAs accumulate in the lung during infection in a strain-dependent manner. Wild−type (**A**), *Rag1^–/–^*, NSG (**B**), and *p47^phox–/–^* (**C**) mice were infected with *A. fumigatus*, and the levels of SCFAs were measured in the lung homogenates at different times post infection by mass spectrometry. * *p* < 0.05; ** *p* < 0.01; *** *p* < 0.001; 3 or 7 vs. 0 dpi. Unpaired *t*−test or one-way ANOVA.

We then evaluated whether SCFAs were produced in vivo by resorting to a murine model of aspergillosis. Mice were infected intranasally (i.n.) with *A. fumigatus* conidia, and a panel of SCFAs was quantified in the lung at different times post infection. As shown in Figure 2A, butyric acid and its isomer isobutyric acid were increased at 7 days post infection along with acetic acid, while propanoic, isovaleric and valeric acids were not increased. These results indicate that *Aspergillus* infection is associated with the production of specific SCFAs.

### 2.2. Manipulating the Composition of the Microbiome or Their Products Alters the Susceptibility to Aspergillus Infection

**Figure 3:**
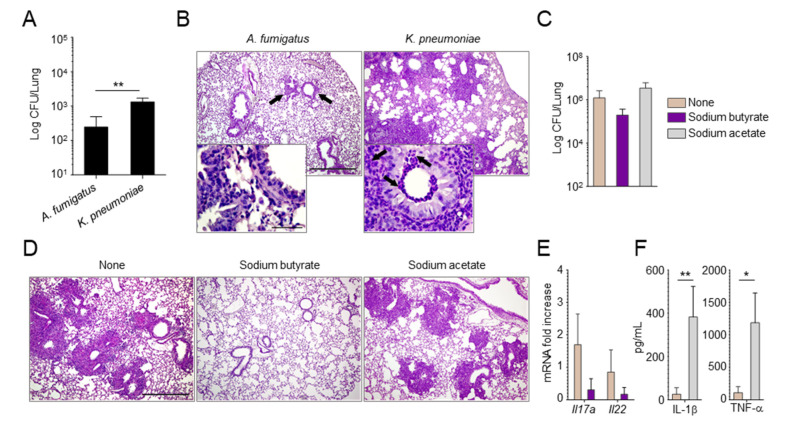
The administration of *Klebsiella pneumoniae* or butyrate has opposite effects in modifying the susceptibility to aspergillosis. Wild−type mice were administered *Klebsiella pneumoniae* (**A**,**B**) or pre-treated with piperacillin–tazobactam and administered butyrate or acetate in the drinking water (**C**–**F**) before intranasal infection with *A. fumigatus* resting conidia. Mice were assessed for fungal growth (Log10 CFU) in the lungs (**A**,**C**), lung histology (periodic acid–Schiff staining, magnification 10× and insets 100×) (**B**,**D**), mRNA levels of *Il17a* and *Il22* (**E**), and protein amounts of IL−1β and TNF−α (**F**) at 5 (**A**,**B**) and 3 days (**C**–**F**) post infection. In B, the black arrows indicate areas of abnormal histopathology and, in the insets, the infiltration of neutrophils. * *p* < 0.05, ** *p* < 0.01, unpaired *t*−test.

Since strictly anaerobic bacteria protected against aspergillosis by preventing the expansion of pathogenic Proteobacteria [17], we evaluated whether tipping the balance between anaerobic bacteria and Proteobacteria could affect the susceptibility to infection. For this purpose, upon administration of the Proteobacteria *Klebsiella pneumoniae* in mice with aspergillosis, we found a higher fungal burden (Figure 3A) and a more severe histopathology (Figure 3B) in *Klebsiella*-exposed mice as compared to wild-type mice, confirming that Proteobacteria may negatively affect the course of the infection.

We then administered butyric acid, as a metabolic product of anaerobic bacteria, in the drinking water of mice before infection with *A. fumigatus* conidia. Mice were pre-treated with piperacillin–tazobactam to lower the basal levels of SCFAs. As a result, we found that butyric acid, while not significantly reducing the fungal burden (Figure 3C), greatly ameliorated lung histopathology (Figure 3D) and reduced the expression level of inflammatory *Il17a* (Figure 3E). In contrast, administration of acetic acid, for which levels were also increased upon infection with *A. fumigatus*, did not play a protective role (Figure 3C,D) and increased the levels of the inflammatory cytokines IL-1β and TNF-α (Figure 3F).

All in all, these results indicate that the composition of the microbiome and the levels of their metabolites play a critical role in the susceptibility to *Aspergillus* infection and are amenable to intervention.

### 2.3. The Composition of the Lung Microbiome and the Levels of SCFAs Are Shaped by the Immune System

**Figure 4:**
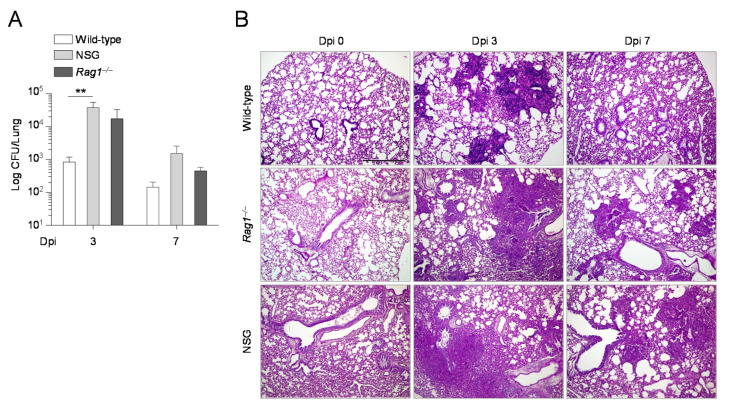
The presence of a dysfunctional immune system affects the susceptibility to aspergillosis. Wild−type, *Rag1^–/–^*, and NSG mice were infected i.n. with *A. fumigatus* resting conidia. Mice were assessed for fungal growth (Log10 CFU) in the lungs (**A**) and lung histology (periodic acid−Schiff staining, representative sections, magnification 10×) (**B**) at 3 and 7 days post infection. Note that the previously reported basal level of inflammation is seen in the lungs of NSG mice [18]. ** *p* < 0.01, two−way ANOVA, Tukey post−test.

Since the composition and function of the microbiota are tuned by the immune system, we resorted to *Rag1^–/–^* mice, deficient in B and T lymphocytes, and NSG mice, which show defects in both the innate and adaptive immune responses, to assess the role of the crosstalk between the microbiota and the immune system in response to Aspergillus. Both *Rag1^–/–^* and NSG mice were more susceptible to infection compared to wild-type mice at 3 days post infection, as shown by increased fungal burden and more severe histopathology (Figure 4). Conversely, at 7 days post infection, while NSG mice were still more susceptible to infection, in agreement with published data [18], *Rag1^–/–^* mice appeared more resistant, as shown by improved histopathology and a trend towards a reduced fungal burden (Figure 4).

**Figure 5:**
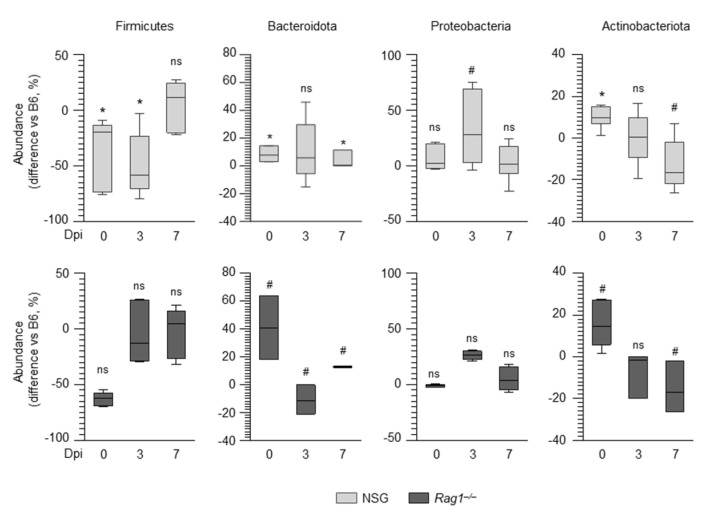
The lung microbiomes of wild−type, *Rag1^–/–^*, and NSG mice display a different composition. Boxplots of the major phyla showing the differences in abundance (expressed as percentage) of NSG and *Rag1^–/–^* mice compared to wild−type mice at each time post infection. Statistical analysis is performed between NSG or *Rag1^–/–^* mice compared to wild−type mice (significance of the zero-mean test on the differences is indicated on top of the corresponding boxplot). ns, not significant; ^#^ *p* < 0.1; * *p* < 0.05.

**Figure 6:**
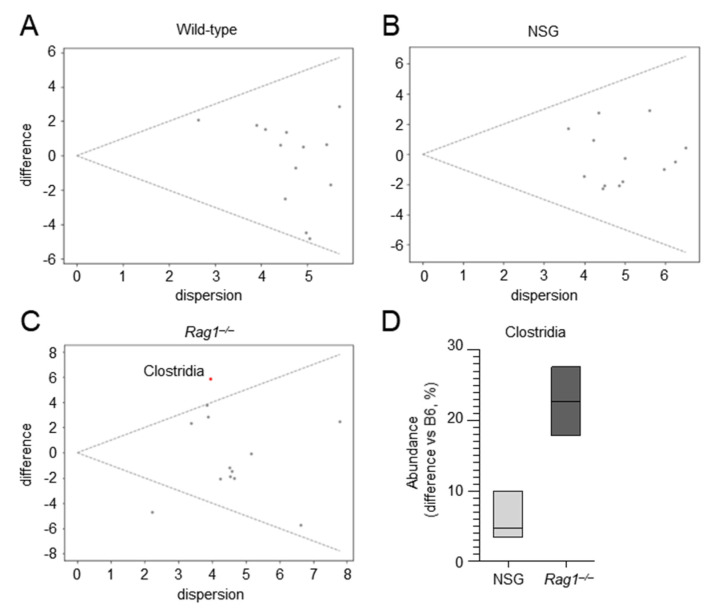
Clostridia are expanded in the lung microbiota of *Rag1^–/–^* mice. MW plot of wild−type (**A**), NSG (**B**), and *Rag1^–/–^* (**C**) data at 0 dpi against the other groups. Differential data were calculated using q2−ALDeX2 applied at the class taxonomic level, and significance was evaluated using the Welch’s t−test. Significant classes are shown in red; non−significant classes are shown as grey dots. (**D**) Boxplots of Clostridia showing the differences in abundance (expressed as percentage) of NSG and *Rag1^–/–^* mice compared to wild−type mice at 0 dpi.

We evaluated the composition of the lung microbiome in these mice and found significant differences in compositional structure at the phylum level between the different groups, as measured by the Jaccard (*p* = 0.007) and Bray–Curtis (*p* = 0.016) indexes. We then compared the abundances of the major phyla in *Rag1^–/–^*, NSG, and wild-type mice at different times post infection. As shown in Figure 5, differences were observed in the composition of lung microbiome at baseline in both *Rag1^–/–^* and NSG mice as compared to wild-type mice. Indeed, both strains had lower levels of Firmicutes, increased levels of Bacteroidota (particularly the *Rag1^–/–^* strain), and similar levels of Proteobacteria. However, when ANOVA-like differential expression (ALDEx) analysis was applied to the lung microbiome of these mice at baseline to identify the classes that significantly differentiate each group from all the others, only one taxa was identified in *Rag1^–/–^* mice, which corresponded to Clostridia, and was found to be expanded as compared to wild-type and NSG mice (Figure 6). Thus, although the baseline levels of Firmicutes were lower, Clostridia were expanded, which would indicate increased levels of strict anaerobes, in *Rag1^–/–^* mice. Of interest, in addition to Clostridia, Bacteroidota were also expanded in *Rag1^–/–^* mice, particularly late in infection, consistent with the failure of Bacteroidota to thrive in the acidic environment generated by the inflammatory response [19]. Thus, the relative expansion of strictly anaerobes through the course of the infection likely limited the expansion of Proteobacteria in Rag1^–/–^ mice as opposed to NSG mice (Figure 5). Therefore, although the underlying genetic immune deficiencies certainly play a role, it can be speculated that the expansion of Proteobacteria in the relative absence of strict anaerobe expansion may contribute to the differing susceptibility of Rag1^–/–^ and NSG mice to infection.

**Figure 7:**
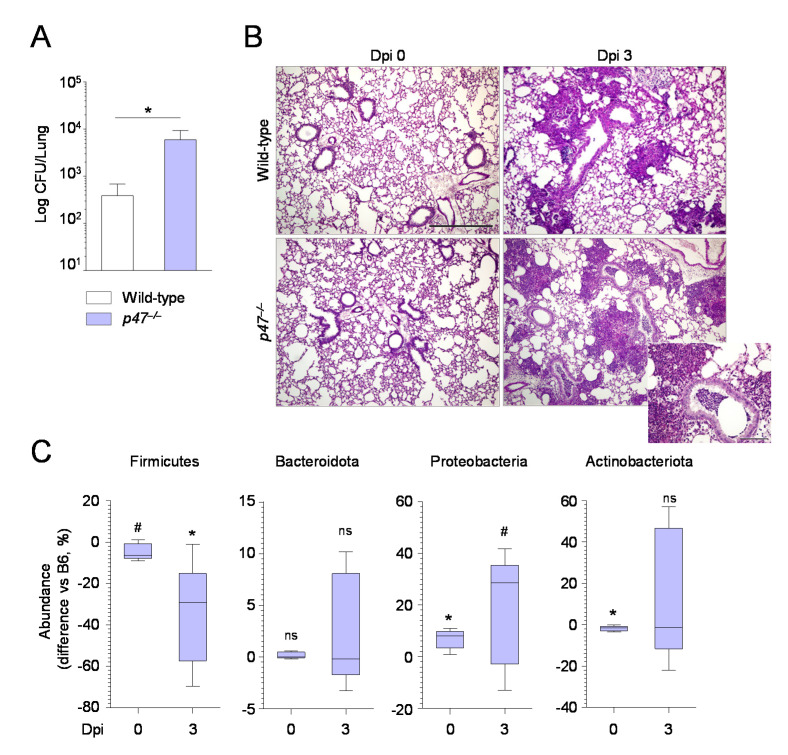
The increased susceptibility of *p47^phox^*^–/–^ mice associates with a dysbiotic microbiome. Wild−type and *p47^phox^*^–/–^ mice were infected i.n. with *A. fumigatus* resting conidia. Mice were assessed for fungal growth (Log10 CFU) in the lungs (**A**), lung histology (periodic acid–Schiff staining, magnification 10×) (**B**) and lung microbiome composition (**C**) at 0 and 3 days post-infection. In (**C**), boxplots of the major phyla show the differences in abundance (expressed as percentage) of *p47^phox^*^–/–^ mice compared to wild−type mice at each time point. Statistical analysis is performed between *p47^phox^*^–/–^ mice compared to wild−type mice (significance of the zero−mean test on the differences is indicated on top of the corresponding boxplot). ns, not significant; ^#^ *p* < 0.1; * *p* < 0.05.

Consistently, on evaluating the levels of SCFA in the lung of those mice, we found that propanoic, butyric, isobutyric, and valeric acids were increased in the lungs of *Rag1^–/–^* mice, while only propanoic acid was slightly increased in NSG mice (Figure 2B). Together, the data show that *Rag1^–/–^*, more than NSG, mice had a microbial configuration similar to that of wild-type mice. Further proving that the expansion of Proteobacteria associated with low abundance of strictly anaerobes may correlate with susceptibility to infection, the results in *p47^phox–/–^* mice, a murine model of chronic granulomatous disease (CGD), showed that these mice, while unable to control the fungal growth (Figure 7A,B), in agreement with previous findings [20], showed increased abundance of Proteobacteria (Figure 7C) and reduced levels of Firmicutes (Figure 7C) and SCFAs (Figure 2B).

Collectively, these results suggest that, although the distinct genetic immune deficiencies play a major role, the expansion of Proteobacteria may contribute to the susceptibility to infection of NSG mice, while the increase in baseline levels of Bacteroidota likely promotes protection in *Rag1^–/–^* mice. Therefore, an intact immune system plays a critical role in shaping the composition of the microbiome in response to *Aspergillus* infection.

## 3. Discussion

The results presented in this study follow our previous findings on the role that the delicate balance between strict anaerobes and Proteobacteria in the lung plays in susceptibility to *Aspergillus* infection [17]. The equilibrium between strict anaerobic bacteria and Proteobacteria is emerging as a common feature in lung disease. Indeed, the healthy lung microbiome is dominated by Bacteroidota, and a decreased abundance is associated with diseased lower airways [21]. Conversely, the increased abundance of Proteobacteria is a common feature in lung dysfunction and disease, for example in asthma, chronic obstructive pulmonary disease, and cystic fibrosis (CF) [19], and is associated with impaired pulmonary function in patients following hematopoietic stem cell transplantation [22]. Thus, the identification of the mechanisms that keep this equilibrium under control holds great potential for therapeutic purposes. Herein, we show that this balance is influenced by the immune system. Indeed, the lung microbiota of *Rag1^–/–^* mice, deficient in the adaptive immune compartment, and NSG mice, bearing multiple defects in the innate and adaptive immune responses, was characterized by an altered balance between strictly anaerobes and Proteobacteria. Increased abundance of strict anaerobes (Clostridia and Bacteroidota) and of Proteobacteria was observed in *Rag1^–/–^* and NSG mice, respectively, with the former showing a microbiome configuration associated with resistance and the latter with susceptibility to the infection, as already reported [17]. Despite being heavily expanded in *Rag1^–/–^* at baseline, the relative contribution, if any, of Actinobacteriota in infection is presently unknown. Similar to NSG mice, *p47^–/–^* mice expanded pathogenic Proteobacteria in the lung during infection, a finding suggesting that the impaired NADPH oxidase and autophagy and increased inflammasome activity, as observed in CGD mice [20], likely represent important innate pathways contributing to the intimate relationship between host immunity and the microbiome.

The influence of the immune system on the composition of the microbiome is not surprising. Indeed, the presence of dysbiosis has been already observed in the gut microbiome in deficiencies of both the innate and adaptive immune compartments [23,24]. However, the possibility of linking specific immune deficiencies to alterations of the lung microbiota associated with a different susceptibility to aspergillosis provides opportunities for personalized interventions in high-risk patients by targeting the defective immune component associated with dysbiosis. For instance, it is known that inflammasomes [25] and autophagy [26] regulate the composition of the gut microbiome with relevance in health and disease. Should this apply to the lung, it implicates that anakinra, the recombinant form of the interleukin-1 receptor antagonist, while dampening inflammasome-dependent inflammation in the lung [20,27], likely restores a local protective microbiome. Along this reasoning, autophagy could similarly be targeted to restore the proper microbiome composition.

This study also suggests that the direct manipulation of the local microbiome could affect the outcome of aspergillosis. Indeed, we show here that the intranasal instillation of the Proteobacteria *Klebsiella pneumonia* increased the susceptibility to infection. This may occur because pneumonia caused by *Klebsiella* exacerbates the inflammatory response, thus creating the conditions for increased fungal growth. Alternatively, *Klebsiella* may directly interact with *Aspergillus*, as recently suggested in an in vitro study [28]. Irrespective of the mechanism(s) involved, while bacteria such as *Klebsiella* may exacerbate fungal pathology, other microbes might play a protective role. This was shown for instance in the protection against pneumococcal colonization upon intranasal administration of *Lactobacillus murinus* [29]. While further studies are needed to determine which, if any, microbial species are associated with protection from *Aspergillus* infection, our study shows that, alternatively, it is possible to resort to microbial metabolites to improve antifungal resistance in the lung, as previously reported for indole-3-carboxaldehyde, produced by the gut microbiota [30] and able to protect from allergic bronchopulmonary aspergillosis [31] and *Aspergillus* pneumonia [17]. In this manuscript, we show that SCFAs are present in the lung during infection. SCFAs have long been recognized for their effects in the gut where they provide an energy source to colonocytes, maintain epithelial barrier integrity, and modulate the immune response [32,33]. Evidence is emerging for a role of SCFAs in the modulation of the physiological and pathological responses in distant organs also, including the brain [34] and the lung [35,36], following their production by the gut microbiota, diffusion via the systemic circulation and engagement of their specific receptors. In our model, we found that butyric acid, but not acetic acid, may have a beneficial effect against aspergillosis, although further studies will be required to determine which SCFAs may hold a therapeutic potential and which mechanisms are involved in the protective effect.

In conclusion, our results indicate that the immune system is required to shape the composition and metabolic potential of the microbiome to allow protection during pulmonary infection, supporting the notion that an alliance between the host and the commensal microbes is critical for the response to pathogen invasion and highlighting the potential therapeutic opportunity of microbial modulation and/or metabolite therapeutics in human infections.

## 4. Materials and Methods

### 4.1. In Vitro Co-Cultures

*A. fumigatus* conidia (Af293) were co-cultured with *Prevotella oris* (DSM 18711) and *Peptostreptococcus anaerobius* (DSM 2949) (purchased from Leibniz-Institut DSMZ-German Collection of Microrganisms and Cell Cultures, Braunschweig, Germany) at a ratio of 1:30 for 24 and 72 h at 37 °C in anaerobic conditions (GenBag, Biomérieux, Hazelwood, WA, USA), and medium was collected for the quantification of isobutyric and butyric acids.

### 4.2. Mice, Infections and Treatments

Eight–to 10–week–old C57BL/6 wild–type mice were purchased from Jackson Laboratories (Bar Harbor, ME, USA). *Rag1^–/–^* and NSG (NOD.Cg-Prkdcscid Il2rgtm1Wjl/SzJ) mice were purchased from Charles River (Calco, Italy). Genetically engineered homozygous *p47^phox–/–^* mice were bred at the Animal Facility of Perugia University, Perugia, Italy. Mice were anesthetized in a plastic cage by inhalation of 3% isoflurane (Abbot Laboratories, North Chicago, IL, USA) in oxygen before intranasal instillation of 2 × 10^7^ *A. fumigatus* resting conidia per 20 μL of saline. Quantification of fungal growth was done as described [37]. For histology, paraffin-embedded sections were stained with periodic acid–Schiff (PAS). In some experiments, butyrate or acetate were administered in drinking water (100 or 150 mM, respectively) in combination with piperacillin–tazobactam (80 mg/kg/day 200μL/ip). For *Klebsiella pneumonia*, the clinical strain was isolated from patient airways, obtained from the Diagnostic Unit of Microbiology, University of Perugia. Bacteria were grown for 12 h to reach the exponential phase. Next, bacteria were pelleted by centrifugation, washed twice with sterile PBS, and the OD of the bacterial suspension was adjusted by spectrophotometry at 600 nm. The intended number of colony forming units CFU (1 × 10^9^/mL) was extrapolated from a standard growth curve. Appropriate dilutions with sterile PBS were made to prepare the inoculum before intranasal aerosol exposure using Biolite stand for mice. Mouse experiments were performed according to Italian Approved Animal Welfare Authorization 360/2015-PR and Legislative Decree 26/2014 regarding the animal license obtained by the Italian Ministry of Health lasting for 5 years (2015–2020).

### 4.3. Real-Time PCR

Real-time PCR was performed using the CFX96 Touch Real-Time PCR detection sys-tem and iTaq Universal SYBR Green Supermix (Bio-Rad, Hercules, CA, USA). Liver was lysed and total RNA was isolated with TRIZOL Reagent (Thermo Fisher Scientific, Waltham, MA, USA) and cDNA was synthesized using the PrimeScript RT Reagent Kit with gDNA Eraser (Takara, Kusatsu, Japan), according to the manufacturer’s instructions. Amplification efficiencies were validated and normalized against β-actin. Each data point was examined for integrity by analysis of the amplification plot. The thermal profile for SYBR Green RT PCR was at 95 °C for 3 min, followed by 40 cycles of denaturation for 30 s at 95 °C, and an annealing/extension step of 30 s at 60 °C. Each da-ta point was examined for integrity by analysis of the amplification plot. The following murine primers were used: *β-actin*: forward AGCCATGTACGTAGCCATCC, reverse CTCTCAGCTGTGGTGGTGAA; *Il17a*: forward GACTACCTCAACCGTTCCAC, reverse CCTCCGCATTGACACAGC; *Il22*: forward CAGCTCAGCTCCTGTCACAT, reverse CAGTTCCCCAATCGCCTTGA.

### 4.4. ELISA

Cytokine levels were determined in lung homogenates by using specific ELISA kits according to the manufacturers’ instructions (R&D System, Minneapolis, MN, USA).

### 4.5. Quantification of SCFA

Supernatants of mice lung homogenates were analyzed by gas chromatography-mass spectrometry (GC-MS) using a 6890 GC and a 5973 A MSD (Agilent Technologies, Milan, Italy) with electron ionization (EI) source. A J&W carbowax GC column (Agilent Technologies) was used, while head space-Solid Phase MicroExtraction (HS-SPME), using a PDMS/CAR/DVB 2 cm fiber, was employed to extract and transfer the analytes in the GC injection port. Deuterium labeled internal standards were used for quantitation.

### 4.6. Metagenomics

DNA was isolated from murine lung using glass beads (Sigma Aldrich, Saint Louis, MO, USA) followed by QIAamp DNA Mini Kit (QIAGEN, Valencia, CA, USA). The bacterial microbiota was evaluated by 16S rRNA. The V3-V4 region of the bacterial 16S genes were sequenced using MiSeq platform (Illumina, San Diego, CA, USA) with primers pair 341F/785R. Sequencing libraries were prepared using NEXTERA XT DNA sample preparation kit (Illumina). Demultiplexing of all libraries for each sequencing lane was accomplished by the Illumina bcl2fastq 2.17.1.14 software. Only reads with at least 100 nucleotides (nt) were retained and then primer sequences were detected, clipped, and oriented into forward-reverse primer orientation. The forward and reverse paired-end reads were imported and analyzed by using Qiime2 platform [38] in a genomic cloud-computing environment based on [39,40] and oriented for biological nano-communication systems in blood vessels for early medical tumor diagnosis [41]. At first, paired-end sequences were denoised, dereplicated, and filtered by any phiX reads and chimera (consensus) by using q2-dada2 quality control method [42] for detecting and correcting (where possible) Illumina amplicon sequence data. In particular, the q2-dada2 method makes use of sequence error profiles to obtain putative error-free sequences, referred to as either sequence variants (SVs) or 100% operational taxonomic units (OTUs). SVs were assigned taxonomy using a Naive Bayes classifier model trained on the Silva138 99% database trimmed to the V3–V4 region of the 16S. The classifier was then applied to the obtained SVs for mapping them to taxonomy. A phylogenetic tree was constructed via sequence alignment with MAFFT [43], filtering the alignment and applying FastTree [44] to generate the tree. The between-samples beta diversity was assessed based on the 16S rRNA, and estimates were calculated on the SVs within QIIME2 using Jaccard and Bray–Curtis distances between samples [45]. An abundance feature table was rarefied according to the lower sample depth (1600 reads) and was aggregated at the phylum level. Significant differences between strains of taxa proportions were evaluated by applying the unpaired non-parametric Wilcoxon test (R v.4.0.2) to differences between the reference group (wild-type) and the strain of interest at a given dpi (0, 3 or 7). The *p*-value was adjusted according to the Hochberg correction. Differences in relative abundances between each strain at a given time-point against all other samples were evaluated by q2-ALDEx2 [46,47]. q2-ALDeX2 was applied to the centered-log ratio transformed data, and significant taxa represented on a MW-plot that shows the fold expression changes (difference) versus the maximum within-condition expression differences (dispersion). A significance level of 0.05 was used, together with the Benjamini–Hochberg corrected *p*-value.

### 4.7. Statistical Analysis

GraphPad Prism software 6.01 (GraphPad Software, San Diego, CA, USA) was used for the analysis. Data are expressed as mean ± SD. Statistical significance was calculated by *t*-test or one-way ANOVA (Bonferroni’s post hoc test) for single and multiple comparisons, respectively.

## Data Availability

The data presented in this study are available on request from the corresponding author.
